# Polar Faculae and the Polar Magnetic Field in Solar Cycles 24 and 25

**DOI:** 10.1007/s11207-026-02702-5

**Published:** 2026-07-15

**Authors:** Julia R. Clark, W. Dean Pesnell, Matthew C. Barzal

**Affiliations:** 1 George Mason University, Fairfax, VA USA; 2 Present Address: Montana State University, Bozeman, MT USA; 3 NASA, Goddard Space Flight Center, Greenbelt, MD USA; 4 Present Address: Arnold, MD USA; 5 University of Maryland, College Park, MD USA

**Keywords:** Polar faculae, Polar magnetic field, Solar cycle

## Abstract

Polar faculae are footprints of the polar magnetic field that are visible as bright spots along intergranular lanes. Unlike equatorial faculae and sunspots, which are found at low to moderate solar latitudes and peak in number at solar maximum, polar faculae are found at latitudes greater than $70^{\circ }$ and peak in number around solar minimum. Polar faculae tend to have the same magnetic polarity as the general polar magnetic field, and their number has been shown to correlate with the strength of that magnetic field. This makes them good candidates for studying the evolution of polar conditions throughout the solar cycle from the ecliptic.

We present a new automated method for counting and studying polar faculae in Helioseismic and Magnetic Imager (HMI) Ic_720s data using a source detection function from the Python library Photutils. We applied this method to both polar regions, using data averaged over each hour throughout the day between 2010 and 2022, the period for which HMI data is available in HelioCloud.

Our results show a variation that is similar to that of the faculae count data from the Debrecen Heliophysical Observatory when they overlap, and extend that time series to December 2022. We also found that the magnetic field of the polar faculae averaged over the polar cap has the same polarity as the polar magnetic field from other measurements. However, we show that faculae with both polarities are present throughout the sunspot cycle. This indicates that some polar faculae may be generated by a local dynamo.

## Introduction

Solar faculae are bright structures in the solar photosphere, most visible near the solar limb, that are associated with accumulations of magnetic flux inside intergranular lanes (Berger et al. 2007). Various models have been proposed to explain the observed properties of faculae. The ‘hot wall’ model suggests that faculae are depressions in the optical surface of the Sun caused by the magnetic field, allowing the observer to see the warmer, brighter walls of the granular upflow (Spruit 1976, 1977; Schatten et al. 1986; Keller et al. 2004). Faculae at lower latitudes are often found near active regions. Such faculae peak in number near solar maximum along with the sunspot number.

In this study, we focus on polar faculae (PFe), which are faculae found poleward of $\pm 70^{\circ}$ latitude. Polar faculae are unique in that they behave independently of active regions and their largest number is observed near solar cycle minimum (Sheeley 1964, 1991). The number of polar faculae has also been shown to have an excellent correlation with the strength of the polar magnetic field (Sheeley 1991). The polar magnetic field is an important component in the evolution of the solar cycle and plays a critical role in solar cycle predictions (e.g., Schatten et al. 1978; Svalgaard, Cliver, and Kamide 2005). As the number of PFe is a good proxy for the polar magnetic field strength and measurements of PFe exist over a longer time than those of the solar magnetic field, PFe have been used to predict the strength of upcoming solar cycles (Makarov, Makarova, and Sivaraman 1989; Makarov and Makarova 1996; Priyal et al. 2014; Janssens 2021). We also anticipate that the motions of PFe could be used in the future to describe plasma velocities in the solar polar regions (Attie et al. 2018; Attie and Innes 2015).

Polar faculae data series have been created by various observatories from the late 1800s to the early 2000s (Makarov and Makarova 1996). A majority of these data sets were created by counting polar faculae in white-light images; however, this is a time-consuming process, especially with the increase in the amount and resolution of satellite data sets.

In this paper, we describe the use of image segmentation in an automated method to identify PFe in HMI data and provide their count and other parameters. We present herein the hourly and daily polar faculae counts from May 1, 2010, to December 31, 2022, and the magnetic flux through individual faculae from specific times per year. We will also compare the variation of PFe counts with those available at the Debrecen Heliophysical Observatory.

## Data and Methods

In this work, we used data from the Helioseismic and Magnetic Imager (HMI) onboard the Solar Dynamics Observatory (SDO) from May 2010 to December 2022 (Schou et al. 2012; Pesnell, Thompson, and Chamberlin 2012).

We are searching for polar faculae in the full-disk continuum intensity data, which is usable as a proxy for white-light images of the photosphere. HMI creates a full-disk continuum intensity image every 45 s by combining images spectrally sampled across the Fe I 6173.3 Å absorption line (Couvidat et al. 2016). Specifically, we are using the 12-min cadence hmi.Ic_nolimbdark_720s series, which are averages of the full-cadence data over a roughly 720 s (12 min) period after the frames are derotated to a central time, resulting in 120 images per day. This averaging process reduces the fluctuations caused by granules, which have a lifetime of 8 – 10 min, and the 5-min variations of the p-modes (Hoeksema et al. 2014; Couvidat et al. 2016). The hmi.Ic_nolimbdark_720s data is produced with limb darkening removed, which allows us to use a constant intensity threshold in our detection algorithm.

Our first attempt at counting polar faculae in the hmi.Ic_nolimbdark_720s series showed a significant amount of periodic noise that varied over the day. For both the northern and southern polar regions, we found that there was a significant increase in polar faculae counted at 12 UTC in the original data. Data products from HMI are known to have 12 h and 24 h variations (Couvidat et al. 2016). We are confident the increase in faculae counted at 12 UTC is a data artifact due to the 24-hour problem, because some of the faculae detected in the data are very short-lived; they only exist in one image. A previous study found that the mean lifespan of a polar faculae is 6.0 hours, with very few polar faculae lasting less than an hour (Hovis-Afflerbach and Pesnell 2022). The cadence of the data is 720s, so true polar faculae should exist across multiple images. To remove the noise, we average the data over an hour period because it is long enough to average out the noise but not dilute the polar faculae.

**Figure 1:**
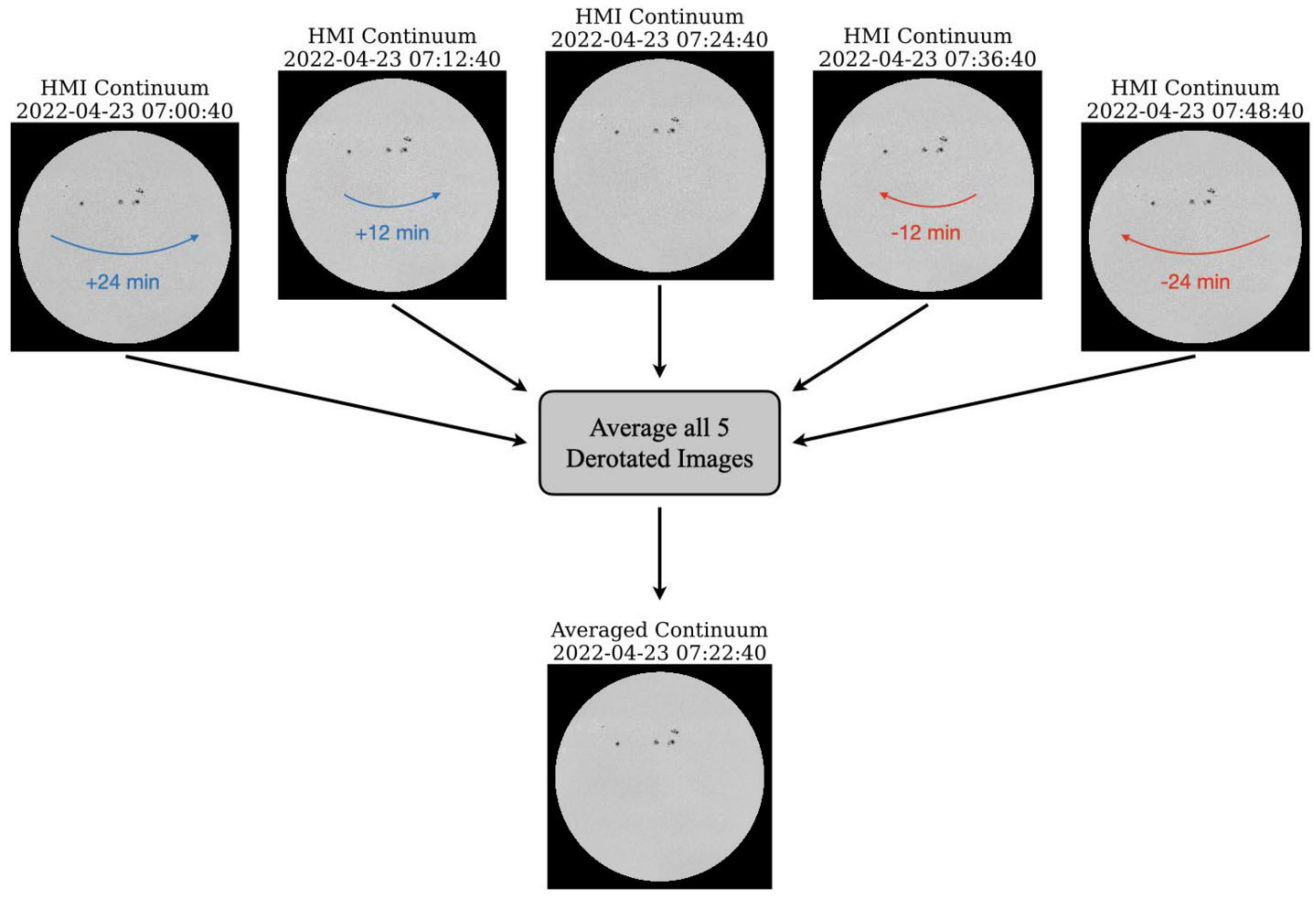
Schematic showing the averaging process. First, the five hmi.Ic_nolimbdark_720s images in an hour are derotated to a single time. The derotated images are then averaged together to create the 3600s data.

We created the hourly averaged data, which we will call the 3600s intensity continuum data, similarly to how the hmi.Ic_720s series is created. This process is depicted in Figure 1. The five 720s images in an hour are derotated to a central time (24 minutes past the hour) and then averaged, resulting in one image per hour. This process is only done if all five images in an hour exist and have good quality.

### HelioCloud

This project was completed using HelioCloud, a cloud computing resource for heliophysics researchers. HelioCloud has a large amount of solar data stored in the cloud, including the SDO data used in this project, which allowed us to quickly access large amounts of data from anywhere without needing to download any files. The HMI images are already centered and rotated such that north-south on the Sun at the central meridian runs in the vertical direction in each image, which reduced the amount of data processing required.

HelioCloud also hosts virtual machines that are user configurable and scalable for larger projects such as this one. There are about 555,240 images in the hmi.Ic_nolimbdark_720s series from May 2010 to December 2022. These images were averaged into about 111,048 3600s images, and PFe were counted in each of those images. Virtual machines are spun up using a web browser interface to provide the computational power to run our code in parallel, significantly reducing the amount of clock time needed to complete this project.

## Detecting Polar Faculae Using Image Segmentation

Our method of automatically counting polar faculae was inspired by an automatic detection code first described in Zhang, Wang, and Liu (2010) to detect active regions in MDI intensitygrams and modified by Muñoz-Jaramillo et al. (2012) to detect polar faculae in the same data after the limb darkening profile was removed.

**Figure 2:**
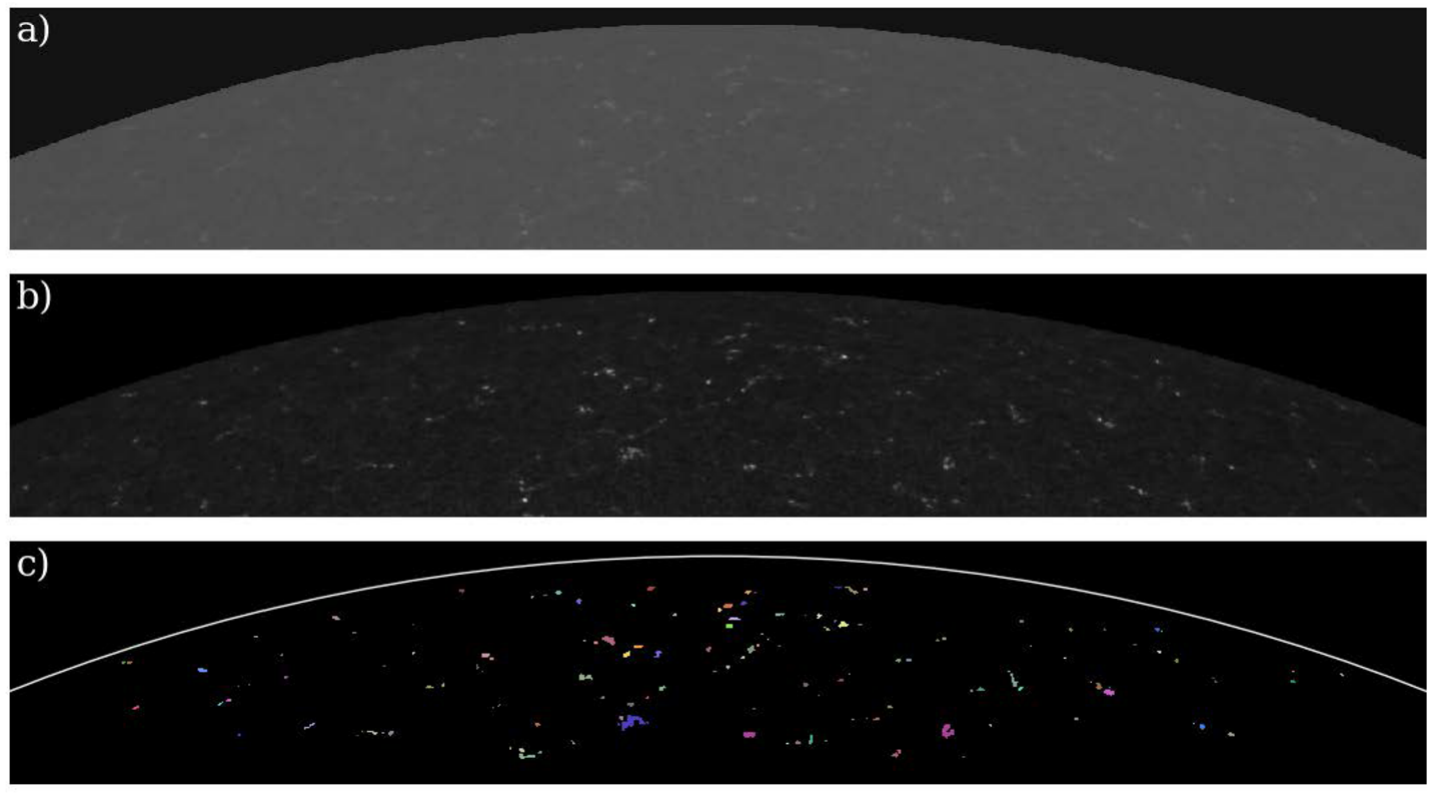
The process of detecting polar faculae starts with the 3600s data shown in a), where polar faculae are 3% brighter than the background. Scaling is applied to each pixel in the 3600s data shown in image b), which enhances the contrast between polar faculae and the background. This scaling maps polar faculae to being brighter than 160. The final step is running image segmentation on the scaled data, resulting in a segmentation image shown in image c). In the segmentation image, each individual polar faculae has a unique integer value, and all background pixels are set to zero. The arc shows the location of the solar limb.

Panel (a) in Figure 2 shows the 3600s data zoomed in on the northern polar region from September 10, 2019, 11:22 UTC. In these data, polar faculae are very faint.

Following Muñoz-Jaramillo et al. (2012), our first step is to apply a scaling of $I_{\mathrm{scaled}} = 100(I)^{15}$ to each pixel in the data. Applying that scaling, as shown in panel (b) of Figure 2, increases contrast and better distinguishes the polar faculae from the background.

Image segmentation is then used on the scaled data to find the polar faculae. We use the detect_sources image segmentation function from Photutils, an affiliated package of Astropy that provides tools for detecting and performing photometry on astronomical sources (Bradley et al. 2023). Although this source detection algorithm was originally used to detect sources in astronomical data, PFe are also low-contrast bright spots on a dark background, and it does a good job of detecting them on the solar disk.

We set the image detection algorithm to detect sources at least two pixels large and with an intensity of at least 160, the same requirements used in Muñoz-Jaramillo et al. (2012). We are using a brightness threshold of 160 because polar faculae are about 3% brighter than the background (Makarov and Makarova 1996). In both MDI and HMI intensitygrams with limb darkening removed, the average background is about 1, making areas brighter than 1.03 polar faculae. With the applied scaling, this value of 1.03 is mapped to about 160. The image segmentation algorithm returns a segmentation image, shown in Figure 2 panel (c), which is an array the same size as the data, where each pixel within each detected faculae is labeled with a positive integer value unique to that source.

Because we are only interested in faculae in the polar regions of the Sun, we did not run image segmentation on the entire solar disk in order to cut down on computation time. Instead, image segmentation was performed on each averaged and scaled image with a mask that covers everything but the northern polar region (above $70^{\circ}$ latitude) and again with a mask that covers everything but the southern polar region (below $-70^{\circ}$ latitude). Both masks also covered the outer pixel edge of the solar limb, which was noisy. The resulting segmentation images were used to count the number of polar faculae in the poles and saved to later measure the magnetic flux in individual faculae.

Once we collected the hourly polar faculae counts in the North and South poles from May 2010 to December 2022, we noticed a significant number of hours with no polar faculae counts and decided to downsample the data by averaging over a day. The gaps in the hourly data were from when HMI data is unavailable due to the Earth blocking SDO’s view of the Sun during its biannual eclipse season, the Moon crossing the Sun at more irregular times, or during instrument calibration maneuvers. Next, we smoothed the data with a Savitzky-Golay filter, with a window length of 25 and a polynomial order of 4, from the Python package SciPy (Savitzky and Golay 1964; Luo, Ying, and Bai 2005; Virtanen et al. 2020). The SciPy implementation of the Savitzky-Golay filter is not robust against NaN values, so the 12 days that had no polar faculae counted were interpolated from neighboring values before the Savitzky-Golay filter was applied to the daily count series.

**Figure 3:**
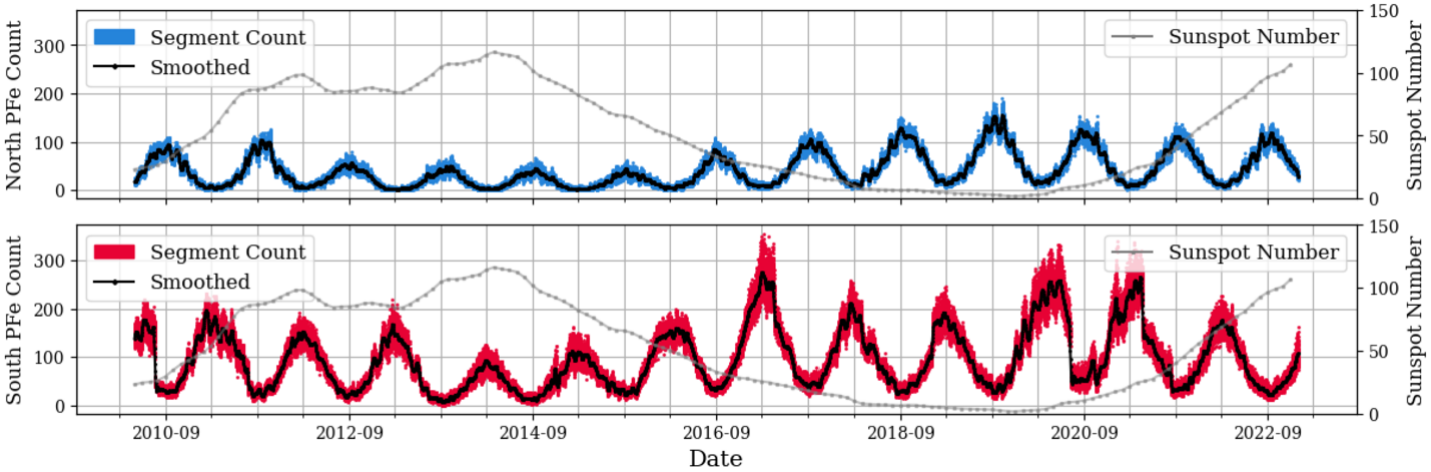
Northern (blue) and Southern (red) hourly polar faculae counts as a function of time starting from the beginning of SDO science data in May 2010 through December 2022. The black lines are the smoothed versions of the underlying data. In April 2014, close to solar maximum, the polar faculae count in the North and South is at its minimum. Close to solar minimum in December 2019, the northern polar faculae count is at its maximum. The southern polar faculae count however peaks in 2017 and again in late 2020. The 13-month smoothed V2.0 Sunspot Number is drawn as a light grey curve and reference to the right scale.

The 3600s PFe counts and the smoothed daily values are plotted in Figure 3, where the Northern and Southern PFe counts are plotted separately. The annual oscillations seen in the data are due to the solar obliquity. In March, the Sun is tilted, so more of the south pole is visible to SDO, and some of the north pole is obscured from view. In September, the opposite is true. This geometric effect results in more northern (southern) polar faculae being visible in September (March) and fewer in March (September).

Previous studies have shown that the variation of the number of polar faculae over time is $180^{\circ}$ out of phase with the sunspot number (Sheeley 1964). Meaning the number of observed polar faculae peaks near solar minimum (when sunspots are at their lowest number) and is lowest at solar maximum (when sunspots appear in the largest amount). This trend can be seen in our data in Figure 3 by comparing the envelope of the PFe counts with the International Sunspot Number (V2.0) drawn in the background. ‘

SDO science data started in 2010, which is 1.4 years after the solar minimum of Solar Cycle 24 in Dec. 2008, so the polar faculae count had already started to decline. Solar maximum occurred in April 2014, around the time both our northern and southern polar counts reached their lowest values. The faculae count then increased until it reached its peak around solar cycle minimum in Dec 2019. Although the northern polar faculae do peak very close to December 2019, we will show below that the southern polar faculae count has a peak in March 2017 and another in late 2020.

We also noticed there are consistently more polar faculae observed overall in the south than in the north. Close to solar minimum, polar faculae counts in the south pole peaked at nearly 300, whereas polar faculae counts in the north pole never exceeded 200. Even at solar maximum, more faculae were observed in the south than in the north.

### Comparison with Debrecen Heliophysics Observatory Data

**Figure 4:**
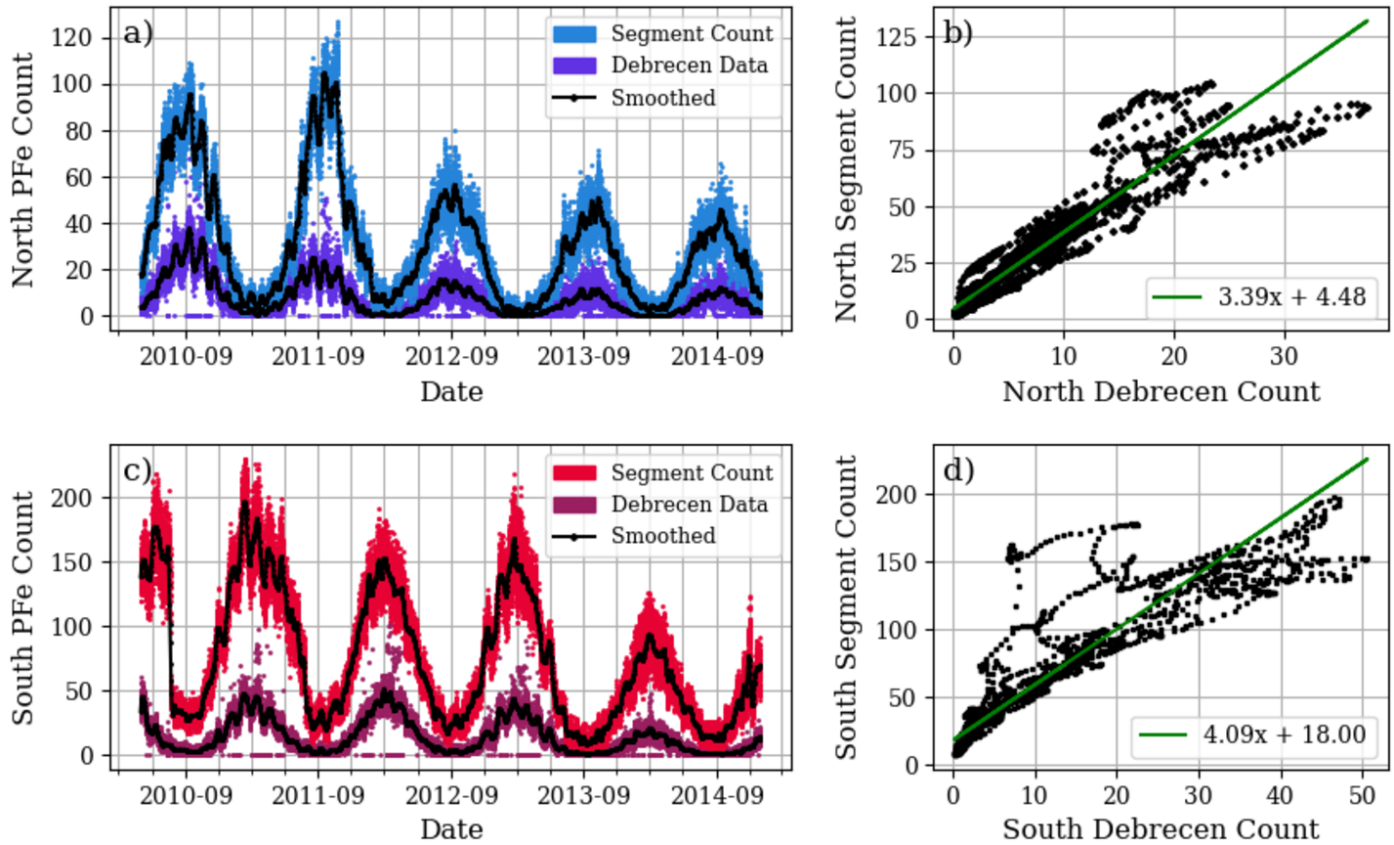
Panel a) shows the hourly northern PFe counts reported by DHO in purple and by our method plotted in blue as a function of time. Panel c) shows the hourly southern PFe counts reported by DHO in magenta and by our method plotted in red as a function of time. The black lines in both panels are the smoothed versions of the underlying data. For both the northern and southern counts our method reports more PFe than the DHO data. Panel b) and d) shows the correlation between the smoothed northern and southern PFe DHO data and our image segmentation method respectively. The green lines in both are fits created using orthogonal distance regression. Our method counts about 3 times as many northern polar faculae and 4 times as many southern polar faculae than DHO.

The Debrecen Heliophysics Observatory (DHO) has hourly faculae data from HMI starting from the beginning of SDO science data in May 2010 through December 2014 (Baranyi, Győri, and Ludmány 2016; Győri, Ludmány, and Baranyi 2017). They counted all faculae on the solar disk in one hmi.Ic_45s image every hour. The location of each faculae was recorded, which allowed us to sort for faculae they found in the northern polar region (above $70^{\circ}$ latitude) and the southern polar region (below $-70^{\circ}$ latitude). The DHO polar faculae count data for each pole was then smoothed using the same method that we used on our count data. A comparison between the DHO polar faculae counts and our counts using image segmentation can be seen in Figure 4.

In Figure 4 panel a) the hourly northern PFe counts reported by DHO are purple and the PFe counts of our image segmentation method are blue. Figure 4 panel c) shows the hourly southern polar faculae counts measured by DHO in magenta and the PFe counts from our image segmentation method in red. The black lines in both graphs are the smoothed versions of the underlying data. Figure 4 panel b) and d) show the correlation between the smoothed DHO data and the smoothed image segmentation data. The green lines are fits to the data created using the orthogonal distance regression model from SciPy (Boggs and Rogers 1990). For the regression model, we used the average count error, which was calculated by averaging the square root of each smoothed data point for both the DHO and our image segmentation PFe counts. These errors are listed in Table 1.

**Table 1:** Fit Coefficients with DHO Counts.

	Assumed Errors		ODR Regression			
	DHO	ImSg	slope	error	Y-int	error
North	2.50	5.14	3.47	0.03	3.84	0.33
South	3.28	8.12	4.02	0.05	19.1	0.87

We can see from Figures 4 a) and 4 c) that our image segmentation method counts more polar faculae overall than DHO in both poles. Looking at the regression lines in graphs b) and d) we can see our method counts about 3.47±0.03 times as many northern polar faculae and 4.02±0.05 times as many southern polar faculae than DHO. It is also interesting to note that both the DHO data and our data show that there are generally more polar faculae observed in the south pole than in the north pole.

A possible reason our method counts more polar faculae than DHO is because we included some of the smaller, dimmer polar faculae in our data set. The image segmentation function we use in our algorithm is dependent on the brightness of the polar faculae and their size. It is likely we are using a lower brightness or size threshold and are including some of the smaller, dimmer polar faculae DHO is excluding.

To test which threshold settings give us similar results to DHO, we found the approximate counts our method would have detected had we independently changed the brightness threshold or size threshold. As described in 3, the segmentation images we used to count PFe can also be used to determine the properties of individual PFe. To test our brightness threshold, we are looking at each polar faculae’s maximum pixel value and counting how many PFe out of our total set would have been bright enough to be detected with different thresholds. Similarly, we are using a polar faculae’s pixel size to count polar faculae that would be large enough to be detected with different size thresholds. This was more efficient than running our detection algorithm multiple times with different threshold settings, because it only requires creating the catalog once regardless of how many threshold values we tested.

For the best comparison, we examine the times when the respective poles are most visible. For northern PFe, this was in August, September, and October, and for southern PFe this was in February, March, and April. To reduce the complexity of the plots, we will examine only five points chosen at random on each day instead of all 24 possible hourly points.

**Figure 5:**
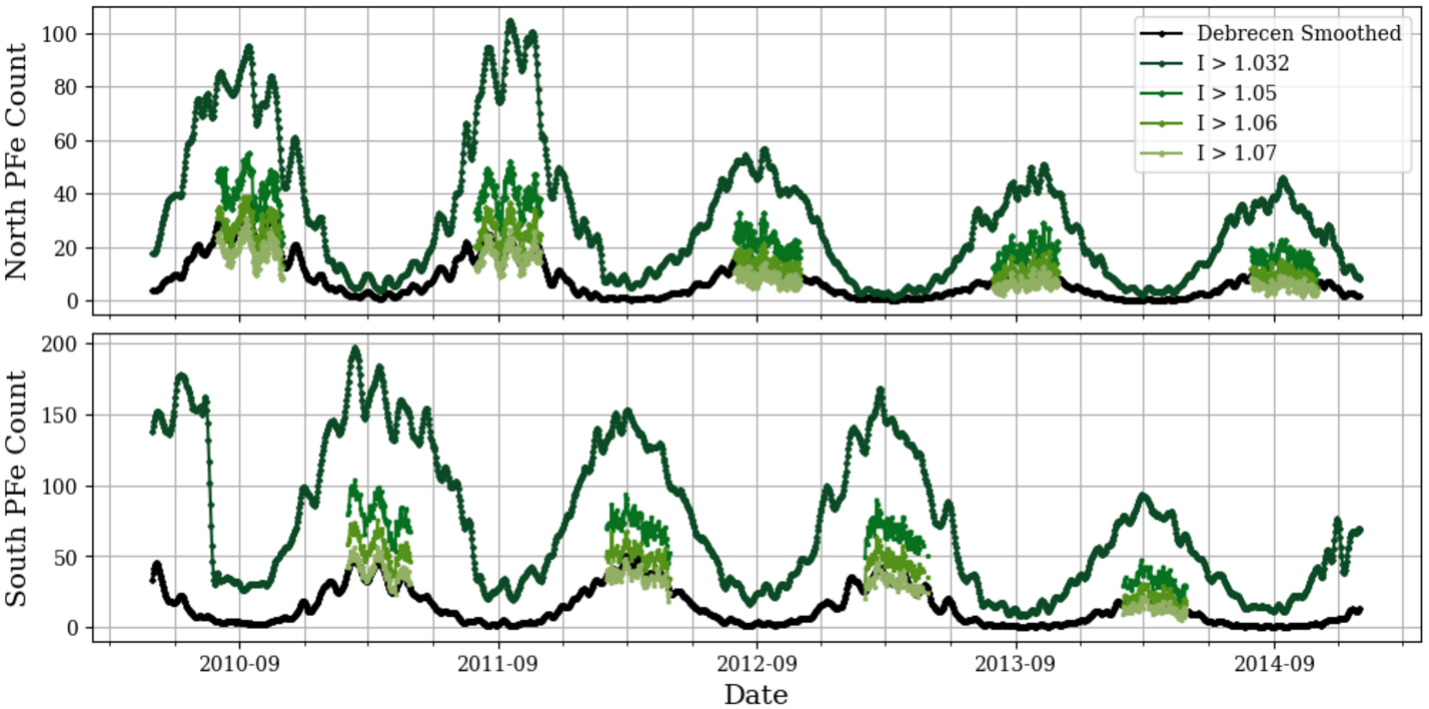
A comparison of current PFe counts in both hemispheres when the intensity threshold used in the current analysis is changed. The baseline threshold is I>1.032. Three other thresholds (I>1.05, 1.06, and 1.07) were used to show that the larger counts in the current set could arise from DHO’s algorithm having a larger intensity threshold.

As expected, Figure 5 shows that the PFe count from image segmentation decreases as the intensity threshold increases. The smoothed DHO data is plotted in black while our data is plotted in various shades of green. Our smoothed image segmentation data is plotted in the darkest green which has a brightness threshold of I=1.032 (with our scaling this maps to a value 160 in the image). The other green lines are the polar faculae counts from our data with increasingly higher intensity thresholds. We found that using a threshold of about 1.06 in the north and 1.07 in the south matched the DHO data fairly well.

**Figure 6:**
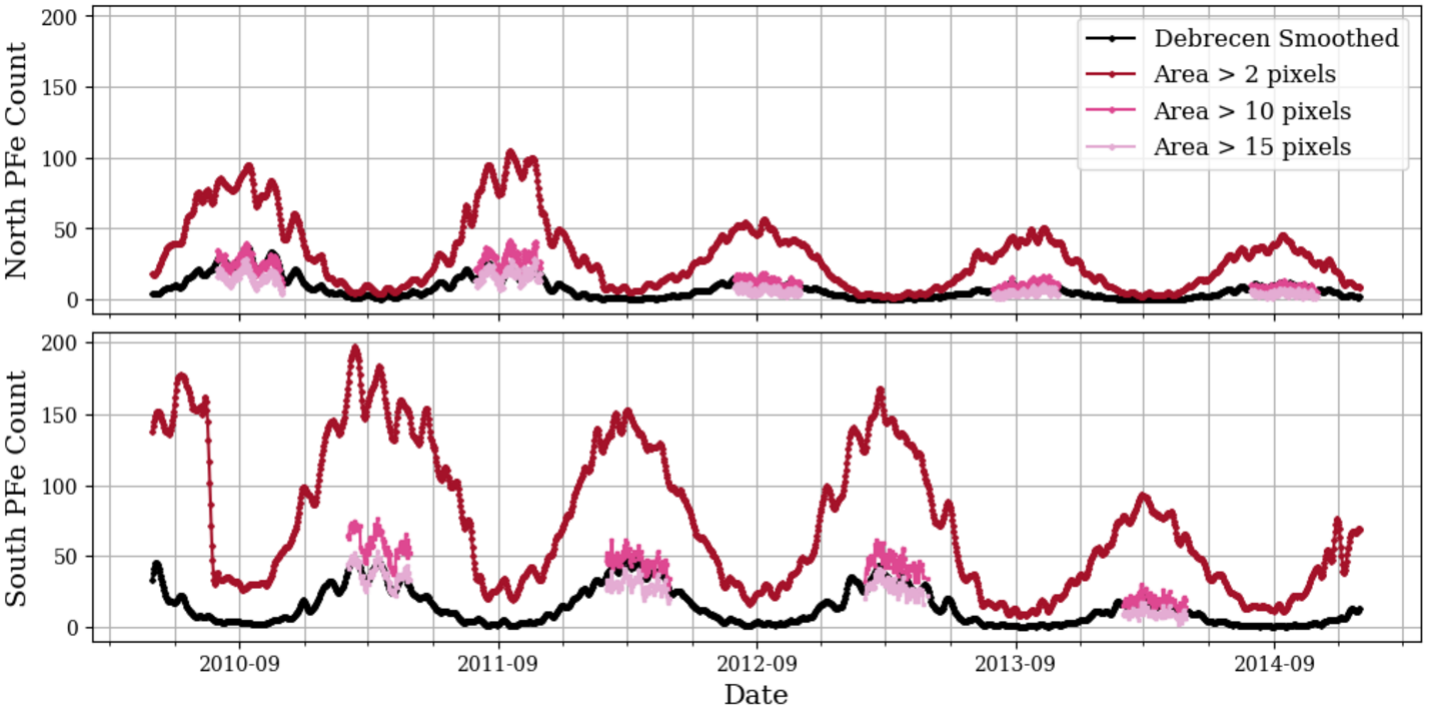
A comparison of current PFe counts in both hemispheres when the required number of contiguous pixels intensity used in the current analysis is changed. A 2-pixel asterism is our baseline threshold. Two other sizes (10 and 15 pixels) were investigated to show that the larger counts in the current set could arise from DHO’s algorithm having a larger size threshold.

Figure 6 shows that increasing the pixel size requirement also decreases the amount of polar faculae counted by our algorithm. The smoothed DHO data is plotted in black while our data is plotted in various shades of red and pink. Our smoothed image segmentation data is plotted in dark red which requires polar faculae to contain at least two pixels (with values greater than 160) in size. The other lighter pink lines are the polar faculae count using a larger size requirement. The southern polar faculae count including only faculae larger than 15 pixels matches fairly well with the DHO data. However the northern polar faculae count including only faculae larger than 10 pixels matches better.

It is interesting that our southern polar faculae counts match the DHO data better with a higher brightness or size threshold than the northern polar faculae count. We don’t expect there to be any differences between counting polar faculae in the north versus the south pole, so it is odd that for our PFe counts to match the DHO analysis different thresholds need to be used. Our regression analysis did show our method counts 4.02±0.05 times as many faculae compared to DHO in the south which is higher than the rate (3.47±0.03) in the North. Our analysis over counting in the South at higher rate than in North could explain why a higher threshold is needed for our counts to match the DHO analysis. There is a larger difference between our data and DHOs in the South compared to the North, so a higher threshold must be used to sort out more faculae. However, it is still unclear why our detection algorithm is over counting faculae at a higher rate in the South than in the North.

From this we conclude that our current analysis is resolving the more numerous faculae that are smaller and lower contrast than the DHO analysis using the hmi.Ic_45s series. Had we chosen a higher intensity threshold or a larger pixel size requirement our data would have matched the DHO count data much better. However, even with the systematic differences our method using image segmentation and the DHO data set do agree and show the same trends and variations in the data.

We are also interested if our analysis and DHO’s agree which years had the most or least PFe counted overall. Looking at the individual peaks of each year, the northern polar faculae data in Figure 4 panel a) show both data sets follow the same general trend that more faculae were observed in 2010 and 2011 compared to 2012 and the subsequent years. The southern polar faculae data in Figure 4 c), shows a similar story where both methods observed a similar trend that from 2010 to 2013 polar faculae were observed in relatively high numbers compared to 2014 where the number of polar faculae dropped.

For a more quantitative comparison, we found the area under the curve using Simpson’s rule from SciPy (Cartwright 2017) for each year to determine which year had the most polar faculae observed for each method. The integrated areas are listed in Table 2.

**Table 2:** Integrated Areas under the PFe Curves.

Year	Northern PFe AUC		Southern PFe AUC	
	DHO	ImSg	DHO	ImSg
2010	4.48 × 10^3^	1.44 × 10^4^	–	–
2011	3.52 × 10^3^	1.47 × 10^4^	6.46 × 10^3^	3.60 × 10^4^
2012	2.28 × 10^3^	1.00 × 10^4^	6.72 × 10^3^	2.86 × 10^4^
2013	1.62 × 10^3^	7.43 × 10^3^	5.05 × 10^3^	2.42 × 10^4^
2014	1.65 × 10^3^	7.07 × 10^3^	2.64 × 10^3^	1.71 × 10^4^

According to the integrated areas, in the north, DHO observed the most polar faculae in 2010 followed by 2011, meanwhile our data found more polar faculae in 2011 than in 2010. After 2012, both sets of data show the number of northern polar faculae observed each year steadily declines. In the south, DHO reported the most polar faculae in 2012, followed by 2011. This disagrees with our analysis which observed more PFe in 2011 than 2012. However, both DHO and our analysis agree 2014 had the lowest counts and 2013 had the second lowest. We excluded data from 2010 in our analysis of southern polar faculae counts because SDO started science data production in May 2010, after the time when the south pole is most visible, so we are missing a significant portion of the peak in this year.

If we take a look at the correlation plots in Figure 4 there are some groups of data points that are significantly above our lines of best fit. In the northern polar faculae plot (b) this group is towards the center of the plot and corresponds to the time from August 2011 to October 2011. In the southern polar faculae plot (d) these outliers are in two groups closer to the middle left of the plot. One of these groups of outliers is from April 2010 to June 2010, and the other is from April 2011 to June 2011. While it is unclear why our analysis is over counting more faculae than our fits predict during these times, it could contribute to why our analysis shows there are more northern polar faculae in 2010 than in 2011 and more southern polar faculae in 2011 than in 2012, while DHO shows the opposite. Other than these two discrepancies both our analysis and DHO agree over which years had the most and least polar faculae.

## Polar Faculae Magnetic Flux over the Solar Cycle

Along with polar faculae counts, we are also interested in looking at the magnetic fields of the polar faculae which have previously been shown to be correlated with the general polar magnetic field (Sheeley 1991). By using the polar faculae as proxies we can examine the evolution of the polar magnetic field over the 12 years of data, which include a complete solar maximum and minimum, along with the rise phase of two sunspot cycles.

As mentioned in Section 3, the segmentation images used to count polar faculae in the 3600s data were saved to measure the magnetic field inside each faculae. Photutils has a SourceCatalog class which creates a catalog of photometry and morphological properties of detected sources. One of the properties available through SourceCatalog is ‘segment flux’, which gives the flux for each individual source defined in a segmentation image. The segmentation image defines which pixels to sum for each polar faculae, which in combination with magnetic field data provides the magnetic flux of our detected polar faculae. Polar faculae range in size, so we divide the total magnetic flux of a faculae by the total number of pixels in a segment to obtain the average magnetic flux through a faculae for better comparison.

The magnetic field data we are using is the hmi.B_720s data series, which are the full-disk vector field data. This data provides the magnetic field in the radial, theta, and phi direction. HMI creates full-disk filtergrams in various wavelengths and polarizations, which are used to determine the Stokes polarization parameters. A Milne-Eddington inversion code is then used to determine the magnetic field from the Stokes parameters (Hoeksema et al. 2014).

**Figure 7:**
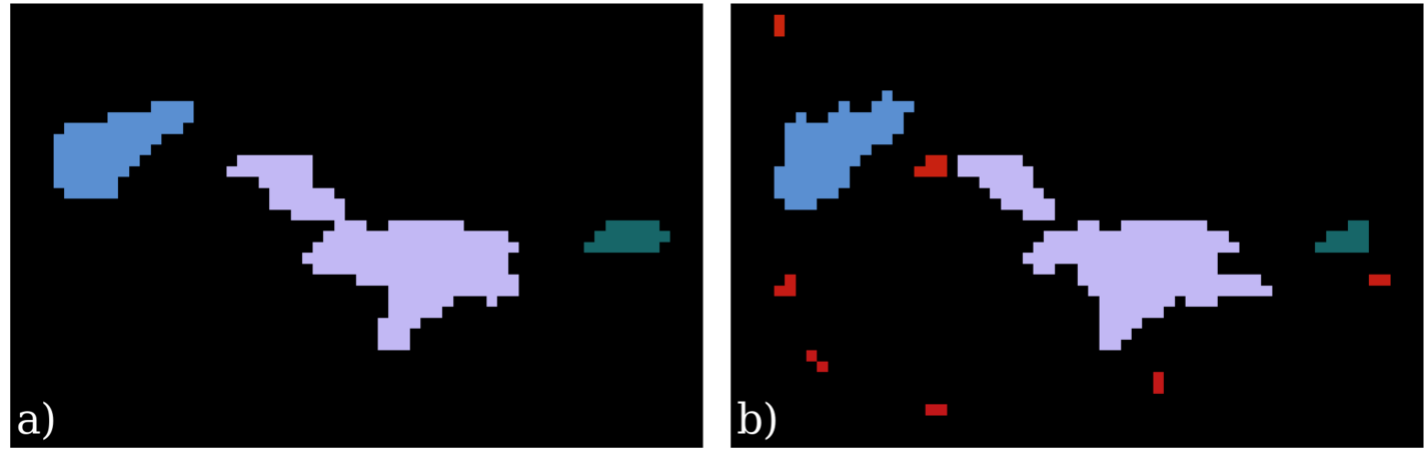
Example of two segmentation images of the same group of polar faculae found in the North pole on September 11, 2019 at 17:22 UTC. Image a) shows the segmentation image from the 3600s averaged intensity continuum data. Image b) shows the segmentation images from the hmi.Ic_nolimbdark_720s. The polar faculae are colored blue, purple, and green in both images, and have some slight differences between the two segmentation images. The red segments in image b) that do not appear in a) are noise.

Conveniently, the hmi.B_720s data are the same size, shape, and resolution as the hmi.Ic_nolimbdark_720s and the averaged 3600s data, so no data processing is necessary. Although we do already have the segmentation images for the 3600s data saved, they do not have great alignment with the 720s data. Figure 7 shows the difference between segmentation images of the 3600s data (right) and the 720s data (left). Although the faculae segments have similar shapes and sizes in both data series, there is disagreement about which pixels are part of a faculae and which are not.

For the best results, we created the polar faculae magnetic flux catalogs using segmentation images from the hmi.Ic_nolimbdark_720s because they have the same cadence as the hmi.B_720s and thus have better alignment. However, as mentioned previously, running image segmentation on the hmi.Ic_nolimbdark_720s picks up noise along with the polar faculae that needs to be filtered out. You can see in Figure 7 that along with the three faculae (colored blue, purple, and green) that appear in the segmentation image from the 3600s (left) and the 720s (right) data there are also multiple small red segments that appear in only the 720s data. These red segments are averaged out in the 3600s data because they are short lived unlike the actual faculae being detected. These small bright points also have about the same magnetic flux as the surrounding background, making them unlikely to be polar faculae.

To sort out the noise, we assumed that only true polar faculae would exist in both the 720s and 3600s segmentation images and that they would likely appear in about the same place. The segments of the two data sets may not overlap exactly, but at least a few pixels should be shared. Therefore, if a segment is a true polar faculae, it should have a minimum pixel value greater than 160 in the scaled 3600s continuum data.

**Figure 8:**
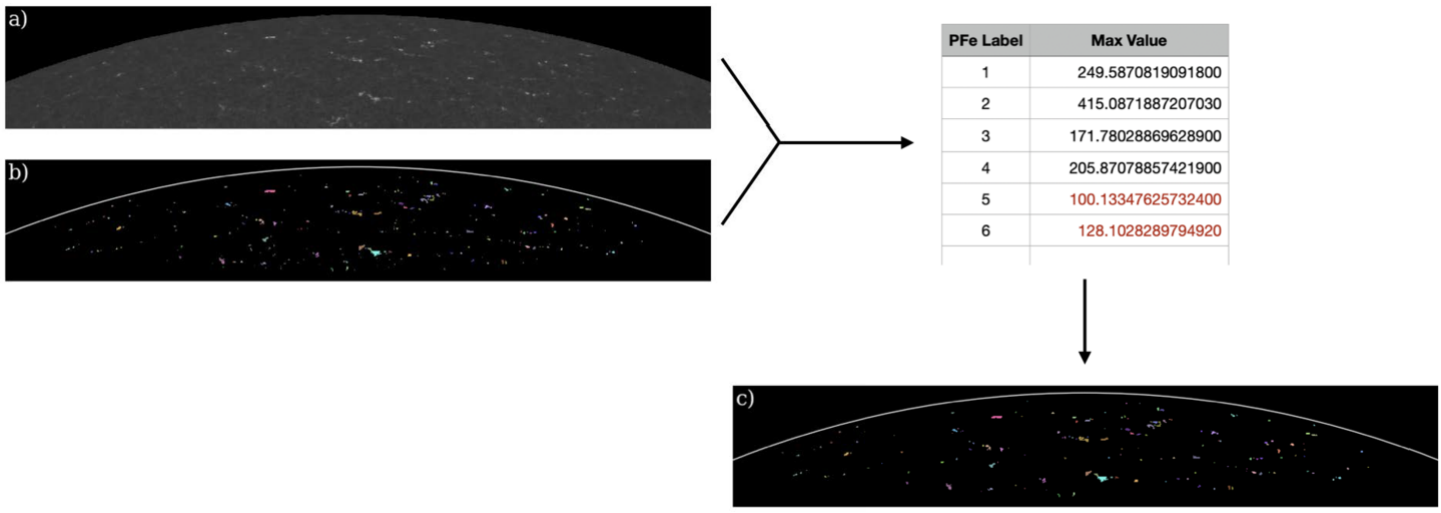
This diagram shows the process of selecting which segments will be used to create the polar faculae catalogs. We start with the scaled 3600s intensity continuum image shown in panel (a) and the segmentation image from the hmi.Ic_nolimbdark_720s image in panel (b). These are then used to create a SourceCatalog providing the label and maximum pixel value of each detected source. We assume that polar faculae should exist in both the 3600s and 720s image with at least a few pixels of overlap, so only sources that have at least one pixel greater than 160 are used to create the polar faculae magnetic flux catalogs. These accepted polar faculae from the 720s data are shown in panel (c).

The process of sorting the noise from the polar faculae is shown in Figure 8. We start by running image segmentation on the hmi.Ic_nolimbdark_720s data. Then a SourceCatalog is created with the 720s segmentation image and the scaled 3600s intensity continuum data providing the label and maximum pixel value of each detected source. We then record the pixel label of sources with maximum pixel values over 160 which are then used to create our polar faculae magnetic flux catalogs.

The final polar faculae catalogs contain the average magnetic flux through each polar faculae detected in an image in the radial, southward, and prograde direction. Note each catalog has information on faculae detected in a single frame of data. To study how the magnetic field of polar faculae changes over the solar cycle we created multiple catalogs each year from 2010 to 2022. Due to time constraints we only created catalogs during the months the poles are most visible. All southern polar faculae catalogs are from the month of March and all northern polar faculae catalogs are from the month of September. We created catalogs for five hours chosen randomly in a day for each day in the month. So there are about 150 northern catalogs and 155 southern catalogs for each year.

### Radial Magnetic Field

The polar faculae catalogs were used to create ridgeplots which show the distributions of the average magnetic flux of polar faculae for each year between 2010 and 2022. The hmi.B_720s data series provides the magnetic field in the radial, θ, and ϕ directions, so we created a separate ridgeplot for each component. All polar faculae in every catalog for a specific year were included in a single distribution so we are plotting the kernel density estimate to make the plots less cluttered and easier to read. The Seaborn Python package was used to create the KDEs for each year’s distribution, assuming a Gaussian kernel was valid (Waskom 2021). These KDEs were then organized in a time sequence to form the ridgeplots below.

**Figure 9:**
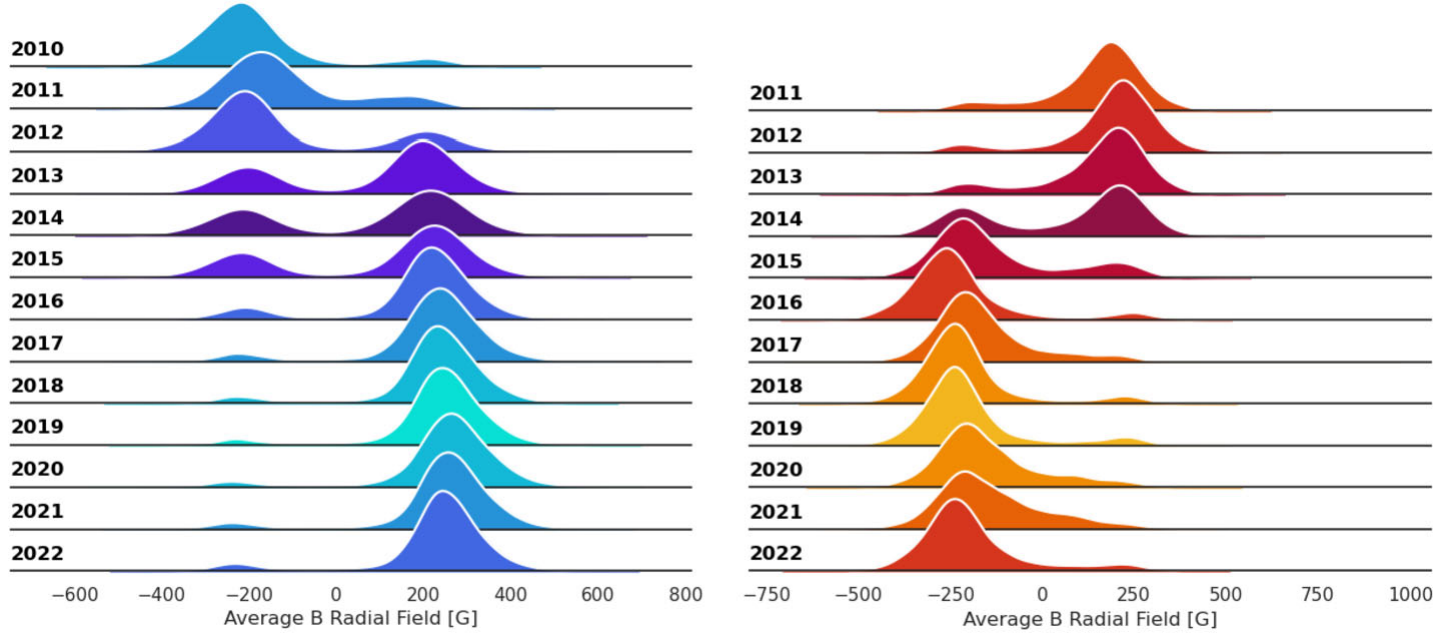
The left-hand panel (in blue) shows the distribution of the average northern polar faculae $B_{r}$ component of the solar magnetic field observed in September of each year from 2010 to 2022. The right-hand panel (in red) shows the distribution of the average southern polar faculae $B_{r}$ component observed in March of each year from 2011 to 2022. Distributions colored with darker shades (dark blue and red) correspond to being close to solar maximum, and lighter shades (light blue and yellow) correspond to being close to solar minimum.

Figure 9 shows the ridgeplot of the average magnetic field flux of PFe in the radial direction. A positive value corresponds to a field pointing outward from the Sun, and a negative value corresponds to a field pointing inwards. The left ridgeplot colored in blue, shows the distributions of northern polar faculae, and the right ridgeplot colored in red, shows the distributions of southern polar faculae. The color of each distribution indicates where that year falls in the solar cycle. Darker shades (dark blue and red) correspond to being close to solar maximum, and lighter shades (light blue and yellow) correspond to being close to solar minimum.

Previous studies have shown that polar faculae have the same polarity as the general polar magnetic field (Homann, Kneer, and Makarov 1997). The ridgeplots show this is true close to solar minimum. In September 2019 the general northern polar field was positive and by March 2019 the general southern polar field was negative (Pishkalo 2019). At these times, nearly all northern polar faculae have a positive radial flux, and nearly all southern polar faculae have a negative radial flux.

At solar cycle maximum, the polar fields go to zero (Sun et al. 2015; Karna, Hess Webber, and Pesnell 2014), so we expected that the magnetic flux of polar faculae would also go to zero at this time. However, our ridge plots show this isn’t the case. Polar faculae that exist at solar maximum have about the same radial field strength as polar faculae that exist at solar minimum. Instead, the ridgeplots show that around solar maximum, polar faculae exist with a mix of both positive and negative polarities, which would still result in a net polar field of about zero. Interestingly, polar faculae seem to always have an average radial magnetic field strength of about 200 to 250 gauss, regardless of when they appear in the solar cycle, which pole they are found in, and their magnetic polarity. This is consistent with the polar magnetic field comprising magnetic elements of several kilogausses in strength (Petrie 2015). The HMI vector field inversions fix the filling factor at unity, effectively reducing the reported field strength to an average of B over the area of each pixel (Hoeksema et al. 2014). Pastor Yabar, Martínez González, and Collados (2018) used Hinode and SoHO/MDI measurements to estimate the filling factor to be ∼0.1−0.2 in the polar regions, which would raise our averages to the expected magnitudes of ∼kG.

Comparing the southern and northern polar faculae ridge plots, it seems that the northern polar faculae switch polarities earlier. In 2012, the majority of northern polar faculae have a positive polarity, but that switches in 2013, where the majority of northern PFe have negative polarity. This switch happens later for the southern polar faculae. In 2014, the majority of southern polar faculae had a positive polarity, and that switched in 2015 where the majority of southern polar faculae had negative polarity.

The minority polarity faculae also seem to remain longer in the north pole than the south pole. After the switch in 2013, the amount of negative polarity polar faculae remaining in the North stays about the same until 2015. However, in the south pole, after the switch in 2015, the amount of polar faculae remaining with positive polarity drops immediately in 2016.

Something to note is that close to solar minimum, the northern polar faculae distributions look very similar year to year and don’t shift left or right from being centered on 250 gauss. However, the southern polar faculae distributions are not as consistent. In 2016, the southern polar faculae distribution is shifted to the left compared to other years’ distributions. In 2017, 2020, and 2021, the distributions are shifted more to the right, slightly shorter, and have more pronounced tails leading past zero. It is worth pointing out that 2017, 2020, and 2021 are also the years with the highest daily counts reported.

### Horizontal Magnetic Field

**Figure 10:**
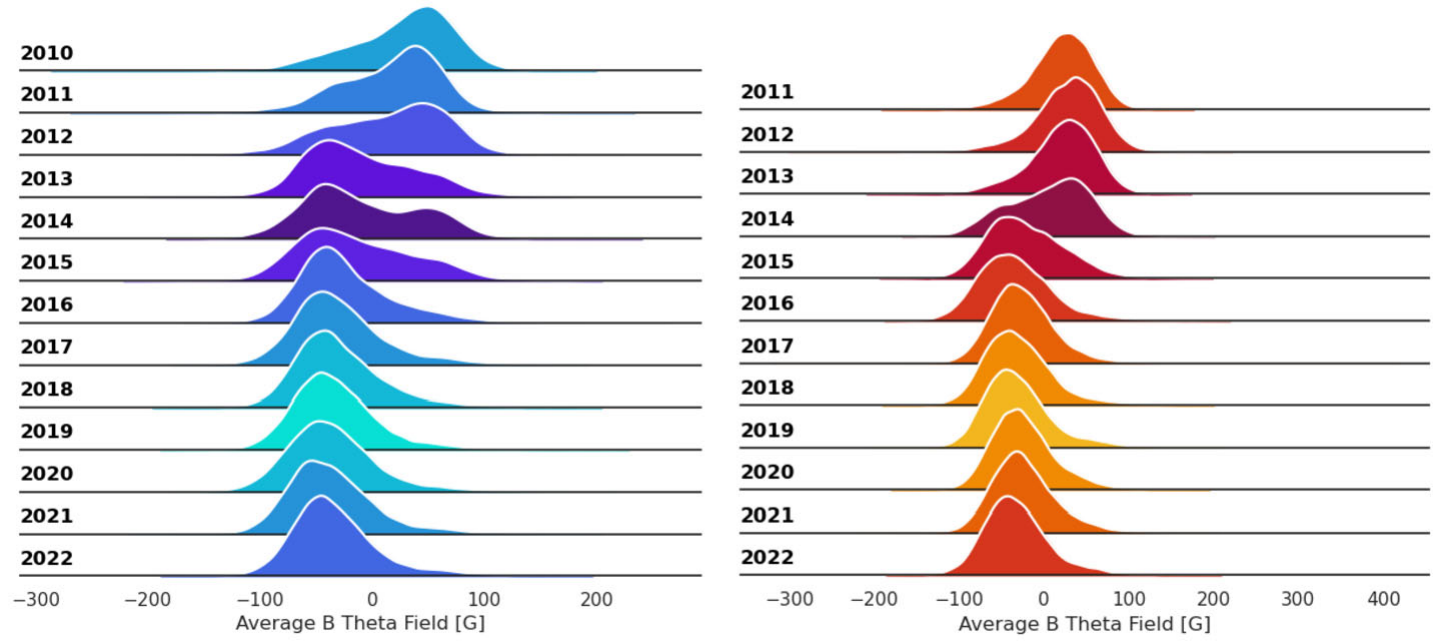
The left-hand panel (in blue) shows the distribution of the average northern polar faculae $B_{\theta}$ component of the magnetic field observed in September of each year from 2010 to 2022. The right-hand panel (in red) shows the distribution of the average southern polar faculae $B_{\theta}$ component observed in March of each year from 2011 to 2022. Distributions colored with darker shades (dark blue and red) correspond to being close to solar maximum, and lighter shades (light blue and yellow) correspond to being close to solar minimum.

**Figure 11:**
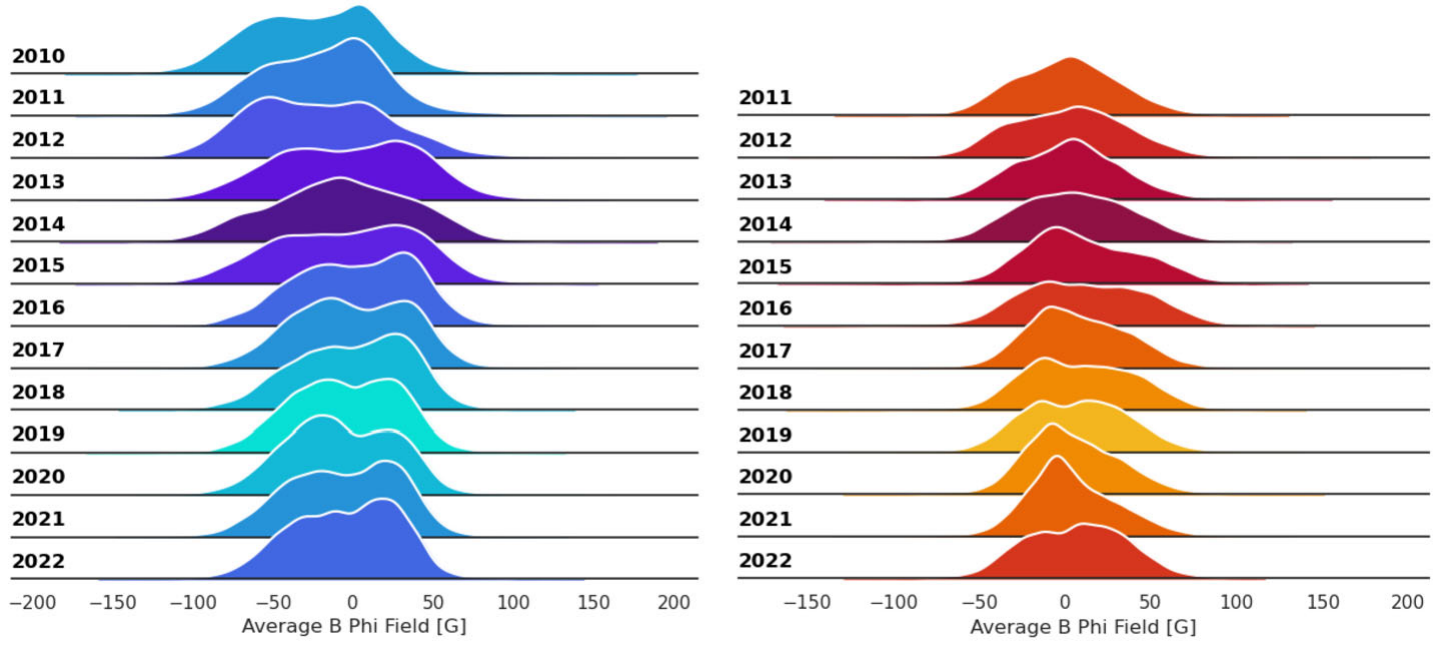
The left-hand panel (in blue) shows the distribution of the average northern polar faculae $B_{\phi}$ component of the magnetic field observed in September of each year from 2010 to 2022. The right-hand panel (in red) shows the distribution of the average southern polar faculae $B_{\phi}$ component observed in March of each year from 2011 to 2022. Distributions colored with darker shades (dark blue and red) correspond to being close to solar maximum, and lighter shades (light blue and yellow) correspond to being close to solar minimum.

Similar ridgeplots were made for polar faculae’s average magnetic field components $B_{\phi}$ (eastward) and $B_{\theta}$ (southward) in Figures 10 and 11 respectively. As before, the left ridgeplots colored in blue show the northern polar faculae distributions and the right ridgeplots colored in red and yellow show the southern polar faculae distributions.

Figure 10 shows that the average $B_{\theta}$ value in polar faculae switches sign around the same time as the average $B_{r}$ field component. From 2011 to 2013, the majority of southern polar faculae have both $B_{\theta }> 0$ and $B_{r}>0$. In 2014 while the majority of PFe have $B_{\theta }> 0$ and $B_{r}>0$ some southern PFe have $B_{\theta }< 0$ and $B_{r} < 0$. After 2015, $B_{\theta }< 0$ and $B_{r} < 0$ for southern PFe.

Similarly, the northern polar faculae have positive $B_{\theta}$ values and negative $B_{r}$ values from 2010 to 2012. From 2013 to 2015, the majority of northern polar faculae have negative $B_{\theta}$ and positive $B_{r}$ values, however some polar faculae remain with positive $B_{\theta}$ and negative $B_{r}$ values. For 2016 and beyond most if not all northern polar faculae have negative $B_{\theta}$ and positive $B_{r}$ values.

Figure 11 shows the distribution of polar faculae’s average $B_{\phi}$ component. Unlike the $B_{\theta}$ and $B_{r}$ components that switch signs over the course of the solar cycle, the $B_{\phi}$ component doesn’t change significantly over the sunspot cycle. The distribution of southern PFe $B_{\phi}$ is centered around zero and weaker than the $B_{\theta}$ and $B_{r}$ components. The largest $B_{\phi}$ values tend to be no greater than ±50 gauss. The $B_{\phi}$ distribution for northern PFe is also, for the most part, centered around zero. However, in 2010 through 2012, the distributions are slightly shifted to the right (i.e., positive values).

These ridgeplots show that polar faculae are closely related to the polar magnetic field. Our data is consistent with previous findings that polar faculae have the same polarity as the general polar magnetic field at solar minimum, and at solar maximum, polar faculae have a mix of positive and negative polarities that results in a net polar magnetic field of zero. Our results also indicate that a majority of the polar magnetic field is consolidated in the polar faculae and that the field is dominated by the radial component.

## Conclusions

This report describes the properties of polar faculae observed in both hemispheres using data from the HMI investigation on SDO. A method of image segmentation was used to detect PFe, which we define as regions of the Sun at high latitudes with contrast greater than 1.03. The result is a dataset of hourly counts spanning from 2010 to 2022.

Our results agree with previous findings that the polar faculae number is correlated with the strength of the polar magnetic field and anti-correlated with the sunspot number. As expected, our Northern and Southern PFe counts were lowest close to solar maximum in April 2014 and were highest close to solar minimum in December 2019. However, our southern PFe counts also have two unexpected peaks in 2017 and late 2020.

When compared to DHO faculae data, our method does count more polar faculae overall. We found that our method counts approximately three times as many northern polar faculae and four times as many southern polar faculae compared to DHO. However, we have shown that this is likely due to our method picking up smaller and lower-contrast PFe. Had we used a higher intensity threshold or excluded smaller faculae, our method would have produced counts much closer to those from DHO. Despite this systematic difference in counts, both our data and the DHO data still show very similar trends.

Along with counting polar faculae, the segmentation algorithm returns the pixels identified as PFe, which are used to extract the vector magnetic field components of individual PFe and provide the first look at the evolution of the polar vector magnetic field over 12 years.

Our results show that close to solar minimum, when the polar magnetic field is strongest, polar faculae exist with one dominant polarity, which is consistent with previous studies. However, during solar maximum, when the polar magnetic field is changing sign, polar faculae exist with both polarities. We also found that the magnetic field in polar faculae doesn’t change in strength over the solar cycle, however it does flip sign. The radial magnetic field of polar faculae stays about 200 G, and the theta component stays about 50 G. The ratio of the radial to the latitudinal magnetic field is about 5 in the Northern hemisphere and 6 in the Southern hemisphere. These ratios show a steeper dependence of $B_{r}$ on $\cos \theta $ than would be expected for a wire-loop magnetic dipole for which $B_{r}/B_{\theta }\sim 2$. These ratios indicate the polar magnetic field is mainly radial and has only a small horizontal component, which is what we expect from earlier research (Svalgaard, Duvall, and Scherrer 1978).

## Data Availability

The SDO data used in this manuscript is available at NASA’s HelioCloud. The Debrecen Heliophysical Observatory data was downloaded from http://fenyi.solarobs.epss.hun-ren.hu/en/databases/SDO/. The V2.0 Sunspot Number was downloaded from the SILSO website at https://sidc.be/SILSO/datafiles. A table of SN extrema to the minimum preceding SC 25 is available at https://sidc.be/SILSO/cyclesminmax.

## Materials Availability

There are no additional materials related to this manuscript.

## Code Availability

All of the calculations and graphics were produced by the authors using Python. This research used version 4.1.4 of the SunPy open source software package (Mumford et al. 2023; The SunPy Community et al. 2020). This work made use of Astropy (http://www.astropy.org), a community-developed core Python package and an ecosystem of tools and resources for astronomy (Robitaille et al. 2013; The Astropy Collaboration et al. 2018, 2022). This research made use of Photutils, an Astropy package for detection and photometry of astronomical sources (Bradley et al. 2023). Any other routines or functions not written by the authors were included from Python’s csv, NumPy, SciPy, and matplotlib libraries.

## References

[CR1] Attie, R., Innes, D.E.: 2015, Magnetic balltracking: tracking the photospheric magnetic flux. *Astron. Astrophys.* **574**, A106. DOI. ADS.

[CR2] Attie, R., Kirk, M.S., Thompson, B.J., Muglach, K., Norton, A.A.: 2018, Precursors of magnetic flux emergence in the moat flows of active region AR12673. *Space Weather* **16**(8), 1143. DOI. ADS.

[CR3] Baranyi, T., Győri, L., Ludmány, A.: 2016, On-line tools for solar data compiled at the debrecen observatory and their extensions with the greenwich sunspot data. *Sol. Phys.* **291**(9–10), 3081. DOI. ADS.

[CR4] Berger, T.E., Voort, L.R.v.d., Löfdahl, M.: 2007, Contrast analysis of solar faculae and magnetic bright points. *Astrophys. J.* **661**(2), 1272. DOI. ADS.

[CR5] Boggs, P.T., Rogers, J.E.: 1990, Orthogonal distance regression. In: Brown, P.J., Fuller, W.A. (eds.) *Statistical Analysis of Measurement Error Models and Applications: Proceedings of the AMS-IMS-SIAM Joint Summer Research Conference Held June 10-16, 1989, with Support from the National Science Foundation and the U.S. Army Research Office*, *Contemporary Mathematics* **112** American Mathematical Society, Providence. ISBN 978-0-8218-5117-3.

[CR6] Bradley, L., Sipőcz, B., Robitaille, T., Tollerud, E., Vinícius, Z., Deil, C., Barbary, K., Wilson, T.J., Busko, I., Donath, A., Günther, H.M., Cara, M., Lim, P.L., Meßlinger, S., Conseil, S., Bostroem, A., Droettboom, M., Bray, E.M., Bratholm, L.A., Barentsen, G., Craig, M., Ginsburg, A., Rathi, S., Pascual, S., Perren, G., Georgiev, I.Y., Kerzendorf, W., Bach, Y.P., Quint, B., Souchereau, H.: 2023, *astropy/photutils: 1.7.0*, Zenodo. DOI. https://zenodo.org/records/7804137.

[CR7] Cartwright, K.: 2017, Simpson’s rule cumulative integration with MS excel and irregularly-spaced data. *J. Math. Sci. Math. Ed.* **12**(2), 1.

[CR8] Couvidat, S., Schou, J., Hoeksema, J.T., Bogart, R.S., Bush, R.I., Duvall, T.L., Liu, Y., Norton, A.A., Scherrer, P.H.: 2016, Observables processing for the helioseismic and magnetic imager instrument on the solar dynamics observatory. *Sol. Phys.* **291**(7), 1887. DOI. ADS. 10.1007/s11207-018-1259-8 PMC6445534 31007294

[CR9] Győri, L., Ludmány, A., Baranyi, T.: 2017, Comparative analysis of Debrecen sunspot catalogues. *Mon. Not. R. Astron. Soc.* **465**(2), 1259. DOI. ADS.

[CR10] Hoeksema, J.T., Liu, Y., Hayashi, K., Sun, X., Schou, J., Couvidat, S., Norton, A., Bobra, M., Centeno, R., Leka, K.D., Barnes, G., Turmon, M.: 2014, The helioseismic and magnetic imager (HMI) vector magnetic field pipeline: overview and performance. *Sol. Phys.* **289**(9), 3483. DOI. ADS. 10.1007/s11207-015-0686-z PMC4456067 26069350

[CR11] Homann, T., Kneer, F., Makarov, V.I.: 1997, Spectro-polarimetry of polar faculae. *Sol. Phys.* **175**(1), 81. DOI. ADS.

[CR12] Hovis-Afflerbach, B., Pesnell, W.D.: 2022, Two new methods for counting and tracking the evolution of polar faculae. *Sol. Phys.* **297**(4), 48. DOI. ADS.

[CR13] Janssens, J.: 2021, Prediction of the amplitude of solar cycle 25 using polar faculae observations. *J. Space Weather Space Clim.* **11**, 3. DOI. ADS.

[CR14] Karna, N., Hess Webber, S.A., Pesnell, W.D.: 2014, Using polar coronal hole area measurements to determine the solar polar magnetic field reversal in solar cycle 24. *Sol. Phys.* **289**, 3381. DOI. ADS.

[CR15] Keller, C.U., Schüssler, M., Vögler, A., Zakharov, V.: 2004, On the origin of solar faculae. *Astrophys. J.* **607**(1), L59. DOI.

[CR16] Luo, J., Ying, K., Bai, L.: 2005, Savitzky-Golay smoothing and differentiation filter for even number data. *Signal Process.* **85**, 1429. DOI.

[CR17] Makarov, V.I., Makarova, V.V.: 1996, Polar faculae and sunspot cycles. *Sol. Phys.* **163**, 267. DOI. ADS.

[CR18] Makarov, V.I., Makarova, V.V., Sivaraman, K.R.: 1989, Do polar faculae on the sun predict a sunspot cycle? *Sol. Phys.* **119**, 45. DOI. ADS.

[CR19] Mumford, S.J., Freij, N., Stansby, D., Christe, S., Ireland, J., Mayer, F., Shih, A.Y., Hughitt, V.K., Ryan, D.F., Liedtke, S., Hayes, L., Pérez-Suárez, D., Vishnunarayan, K.I., Barnes, W., Chakraborty, P., Inglis, A., Pattnaik, P., Sipőcz, B., MacBride, C., Sharma, R., Leonard, A., Hewett, R., Hamilton, A., Manhas, A., Panda, A., Earnshaw, M., Choudhary, N., Kumar, A., Singh, R., Chanda, P., Haque, M.A., Kirk, M.S., Mueller, M., Konge, S., Srivastava, R., Wentzel-Long, M., Jain, Y., Bennett, S., Baruah, A., Arbolante, Q., Charlton, M., Maloney, S., Mishra, S., Paul, J.A., Verma, A., Chorley, N., Chouhan, A., Himanshu, M.J.P., Zivadinovic, L., Modi, S., Sharma, Y., Naman9639, B.M.G., Rozo, J.I.C., Manley, L., Ivashkiv, K., Laitinen, T., Chatterjee, A., Forstner, J.F.v., Bazán, J., Stern, K.A., Gieseler, J., Evans, J., Jain, S., Malocha, M., Ghosh, S., Airmansmith, Stańczak, D., Singh, R.R., Visscher, R.D., Verma, S., SophieLemos, A.A., Alam, A., Buddhika, D., Pathak, H., Rideout, J.R., Sharma, S., Park, J., Bates, M., Wilson, A., Shukla, D., Giger, M., Mishra, P., Sharma, D., Goel, D., Taylor, G., Cetusic, G., Reiter, G., Jacob, I.M., Dacie, S., Dubey, S., Eigenbrot, A., Bray, E.M., Surve, R., Zahniy, S., Sidhu, S., Meszaros, T., Parkhi, U., Russell, W., Bose, A., Pandey, A., Price-Whelan Amogh, A., Chicrala, A., Ankit, G.C., D’Avella, D., Williams, D., Verma, D., Ballew, J., Agrawal, K., Murphy, N., Lodha, P., Robitaille, T., Augspurger, T., Krishan, Y., honey, neerajkulk, Bhope, A., Gaba, A.S., Hill, A., Mampaey, B., Wiedemann, B.M., Molina, C., Briseno, D.G., Keşkek, D., Habib, I., Letts, J., Singaravelan, K., Ranjan, K., Altunian, N., Streicher, O., Gomillion, R., Agarwal, S., Kothari, Y., Nomiya, Y., mridulpandey, Stevens, A.L., Abijith, B., Bahuleyan, A., Kaszynski, A., Alex, W., Mehrotra, A., Tang, A., Sinha, A., Smith, A., Kustov, A., Stone, B., Bard, C., Arias, E., Tollerud, E., Dover, F.M., Verstringe, F., Kumar, G., Mathur, H., Babuschkin, I., Calixto, J., Wimbish, J., Qing, J., Buitrago-Casas, J.C., Krishna, K., Chaudhari, K., Hiware, K., Ghosh, K., Lyes, M.M., Mangaonkar, M., Cheung, M., Mendero, M., Dedhia, M., Schoentgen, M., Shahdadpuri, N., Srinivasan, N., Gyenge, N.G., Mekala, R.R., Das, R., Mishra, R., Sharma, R., Srikanth, S., Jain, S., Kannojia, S., Yadav, T., Paul, T., Wilkinson, T.D., Caswell, T.A., Braccia, T., Pereira, T.M.D., Gates, T., Dang, T.K., Bankar, V., Jamieson, W., Agrawal, Y., platipo, resakra, tal66, yasintoda, Attie, R., Murray, S.A.: 2023, *SunPy*, Zenodo. DOI. https://zenodo.org/records/7641693.

[CR20] Muñoz-Jaramillo, A., Sheeley, N.R., Zhang, J., DeLuca, E.E.: 2012, Calibrating 100 years of polar faculae measurements: implications for the evolution of the heliospheric magnetic field. *Astrophys. J.* **753**(2), 146. DOI. ADS.

[CR21] Pastor Yabar, A., Martínez González, M.J., Collados, M.: 2018, Magnetic topology of the North solar pole. *Astron. Astrophys.* **616**, A46. DOI. ADS.

[CR22] Pesnell, W.D., Thompson, B.J., Chamberlin, P.C.: 2012, The solar dynamics observatory (SDO). *Sol. Phys.* **275**(1), 3. DOI.

[CR23] Petrie, G.J.D.: 2015, Solar magnetism in the polar regions. *Living Rev. Sol. Phys.* **12**(1), 5. DOI. ADS.

[CR24] Pishkalo, M.I.: 2019, On polar magnetic field reversal in solar cycles 21, 22, 23, and 24. *Sol. Phys.* **294**(10), 137. DOI. ADS.

[CR25] Priyal, M., Banerjee, D., Karak, B.B., Muñoz-Jaramillo, A., Ravindra, B., Choudhuri, A.R., Singh, J.: 2014, Polar network index as a magnetic proxy for the solar cycle studies. *Astrophys. J. Lett.* **793**(1), L4. DOI.

[CR26] Robitaille, T.P., Tollerud, E.J., Greenfield, P., Droettboom, M., Bray, E., Aldcroft, T., Davis, M., Ginsburg, A., Price-Whelan, A.M., Kerzendorf, W.E., Conley, A., Crighton, N., Barbary, K., Muna, D., Ferguson, H., Grollier, F., Parikh, M.M., Nair, P.H., Günther, H.M., Deil, C., Woillez, J., Conseil, S., Kramer, R., Turner, J.E.H., Singer, L., Fox, R., Weaver, B.A., Zabalza, V., Edwards, Z.I., Bostroem, K.A., Burke, D.J., Casey, A.R., Crawford, S.M., Dencheva, N., Ely, J., Jenness, T., Labrie, K., Lim, P.L., Pierfederici, F., Pontzen, A., Ptak, A., Refsdal, B., Servillat, M., Streicher, O.: 2013, Astropy: a community python package for astronomy. *Astron. Astrophys.* **558**, A33. DOI.

[CR27] Savitzky, A., Golay, M.J.E.: 1964, Smoothing and differentiation of data by simplified least squares procedures. *Anal. Chem.* **36**, 1627. DOI. ADS. 10.1021/ac60319a045 22324618

[CR28] Schatten, K.H., Scherrer, P.H., Svalgaard, L., Wilcox, J.M.: 1978, Using dynamo theory to predict the sunspot number during solar cycle 21. *Geophys. Res. Lett.* **5**, 411. DOI. ADS.

[CR29] Schatten, K.H., Mayr, H.G., Omidvar, K., Maier, E.: 1986, A hillock and cloud model for faculae. *Astrophys. J.* **311**, 460. DOI. ADS.

[CR30] Schou, J., Scherrer, P.H., Bush, R.I., Wachter, R., Couvidat, S., Rabello-Soares, M.C., Bogart, R.S., Hoeksema, J.T., Liu, Y., Duvall, T.L., Akin, D.J., Allard, B.A., Miles, J.W., Rairden, R., Shine, R.A., Tarbell, T.D., Title, A.M., Wolfson, C.J., Elmore, D.F., Norton, A.A., Tomczyk, S.: 2012, Design and ground calibration of the helioseismic and magnetic imager (HMI) instrument on the solar dynamics observatory (SDO). *Sol. Phys.* **275**(1–2), 229. DOI. ADS.

[CR31] Sheeley, N.R. Jr.: 1964, Polar faculae during the sunspot cycle. *Astrophys. J.* **140**, 731. DOI. ADS.

[CR32] Sheeley, N.R. Jr.: 1991, Polar faculae: 1906–1990. *Astrophys. J.* **374**, 386. DOI. ADS.

[CR33] Spruit, H.C.: 1976, Pressure equilibrium and energy balance of small photospheric fluxtubes. *Sol. Phys.* **50**, 269. DOI. ADS.

[CR34] Spruit, H.C.: 1977, Heat flow near obstacles in the solar convection zone. *Sol. Phys.* **55**, 3. DOI. ADS.

[CR35] Sun, X., Hoeksema, J.T., Liu, Y., Zhao, J.: 2015, On polar magnetic field reversal and surface flux transport during solar cycle 24. *Astrophys. J.* **798**(2), 114. DOI. ADS.

[CR36] Svalgaard, L., Cliver, E.W., Kamide, Y.: 2005, Sunspot cycle 24: Smallest cycle in 100 years? *Geophys. Res. Lett.* 32(1). DOI. ADS.

[CR37] Svalgaard, L., Duvall, T.L. Jr., Scherrer, P.H.: 1978, The strength of the Sun’s polar fields. *Sol. Phys.* **58**(2), 225. DOI. ADS.

[CR38] The Astropy Collaboration, Price-Whelan, A.M., Sipőcz, B.M., Günther, H.M., Lim, P.L., Crawford, S.M., Conseil, S., Shupe, D.L., Craig, M.W., Dencheva, N., Ginsburg, A., VanderPlas, J.T., Bradley, L.D., Pérez-Suárez, D., de Val-Borro, M., Aldcroft, T.L., Cruz, K.L., Robitaille, T.P., Tollerud, E.J., Ardelean, C., Babej, T., Bachetti, M., Bakanov, A.V., Bamford, S.P., Barentsen, G., Barmby, P., Baumbach, A., Berry, K.L., Biscani, F., Boquien, M., Bostroem, K.A., Bouma, L.G., Brammer, G.B., Bray, E.M., Breytenbach, H., Buddelmeijer, H., Burke, D.J., Calderone, G., Rodríguez, J.L.C., Cara, M., Cardoso, J.V.M., Cheedella, S., Copin, Y., Crichton, D., DÁvella, D., Deil, C., Depagne, É., Dietrich, J.P., Donath, A., Droettboom, M., Earl, N., Erben, T., Fabbro, S., Ferreira, L.A., Finethy, T., Fox, R.T., Garrison, L.H., Gibbons, S.L.J., Goldstein, D.A., Gommers, R., Greco, J.P., Greenfield, P., Groener, A.M., Grollier, F., Hagen, A., Hirst, P., Homeier, D., Horton, A.J., Hosseinzadeh, G., Hu, L., Hunkeler, J.S., Ivezić, Ž., Jain, A., Jenness, T., Kanarek, G., Kendrew, S., Kern, N.S., Kerzendorf, W.E., Khvalko, A., King, J., Kirkby, D., Kulkarni, A.M., Kumar, A., Lee, A., Lenz, D., Littlefair, S.P., Ma, Z., Macleod, D.M., Mastropietro, M., McCully, C., Montagnac, S., Morris, B.M., Mueller, M., Mumford, S.J., Muna, D., Murphy, N.A., Nelson, S., Nguyen, G.H., Ninan, J.P., Nöthe, M., Ogaz, S., Oh, S., Parejko, J.K., Parley, N., Pascual, S., Patil, R., Patil, A.A., Plunkett, A.L., Prochaska, J.X., Rastogi, T., Janga, V.R., Sabater, J., Sakurikar, P., Seifert, M., Sherbert, L.E., Sherwood-Taylor, H., Shih, A.Y., Sick, J., Silbiger, M.T., Singanamalla, S., Singer, L.P., Sladen, P.H., Sooley, K.A., Sornarajah, S., Streicher, O., Teuben, P., Thomas, S.W., Tremblay, G.R., Turner, J.E.H., Terrón, V., van Kerkwijk, M.H., de la Vega, A., Watkins, L.L., Weaver, B.A., Whitmore, J.B., Woillez, J., Zabalza, V.: 2018, The astropy project: building an inclusive, open-science project and status of the v2.0 core package. *Astron. J.* **156**(3), 123. DOI. ADS.

[CR39] The Astropy Collaboration, Price-Whelan, A.M., Lim, P.L., Earl, N., Starkman, N., Bradley, L., Shupe, D.L., Patil, A.A., Corrales, L., Brasseur, C.E., Nöthe, M., Donath, A., Tollerud, E., Morris, B.M., Ginsburg, A., Vaher, E., Weaver, B.A., Tocknell, J., Jamieson, W., van Kerkwijk, M.H., Robitaille, T.P., Merry, B., Bachetti, M., Günther, H.M., Aldcroft, T.L., Alvarado-Montes, J.A., Archibald, A.M., Bódi, A., Bapat, S., Barentsen, G., Bazán, J., Biswas, M., Boquien, M., Burke, D.J., Cara, D., Cara, M., Conroy, K.E., Conseil, S., Craig, M.W., Cross, R.M., Cruz, K.L., D’Eugenio, F., Dencheva, N., Devillepoix, H.A.R., Dietrich, J.P., Eigenbrot, A.D., Erben, T., Ferreira, L., Foreman-Mackey, D., Fox, R., Freij, N., Garg, S., Geda, R., Glattly, L., Gondhalekar, Y., Gordon, K.D., Grant, D., Greenfield, P., Groener, A.M., Guest, S., Gurovich, S., Handberg, R., Hart, A., Hatfield-Dodds, Z., Homeier, D., Hosseinzadeh, G., Jenness, T., Jones, C.K., Joseph, P., Kalmbach, J.B., Karamehmetoglu, E., Kałuszyński, M., Kelley, M.S.P., Kern, N., Kerzendorf, W.E., Koch, E.W., Kulumani, S., Lee, A., Ly, C., Ma, Z., MacBride, C., Maljaars, J.M., Muna, D., Murphy, N.A., Norman, H., O’Steen, R., Oman, K.A., Pacifici, C., Pascual, S., Pascual-Granado, J., Patil, R.R., Perren, G.I., Pickering, T.E., Rastogi, T., Roulston, B.R., Ryan, D.F., Rykoff, E.S., Sabater, J., Sakurikar, P., Salgado, J., Sanghi, A., Saunders, N., Savchenko, V., Schwardt, L., Seifert-Eckert, M., Shih, A.Y., Jain, A.S., Shukla, G., Sick, J., Simpson, C., Singanamalla, S., Singer, L.P., Singhal, J., Sinha, M., Sipőcz, B.M., Spitler, L.R., Stansby, D., Streicher, O., Šumak, J., Swinbank, J.D., Taranu, D.S., Tewary, N., Tremblay, G.R., de Val-Borro, M., Van Kooten, S.J., Vasović, Z., Verma, S., Cardoso, J.V.d.M., Williams, P.K.G., Wilson, T.J., Winkel, B., Wood-Vasey, W.M., Xue, R., Yoachim, P., Zhang, C., Zonca, A.: 2022, The astropy project: sustaining and growing a community-oriented open-source project and the latest major release (v5.0) of the core package. *Astrophys. J.* **935**(2), 167. DOI. ADS.

[CR40] The SunPy Community, Barnes, W.T., Bobra, M.G., Christe, S.D., Freij, N., Hayes, L.A., Ireland, J., Mumford, S., Perez-Suarez, D., Ryan, D.F., Shih, A.Y., Contributors, P.P., Chanda, P., Glogowski, K., Hewett, R., Hughitt, V.K., Hill, A., Hiware, K., Inglis, A., Kirk, M.S.F., Konge, S., Mason, J.P., Maloney, S.A., Murray, S.A., Panda, A., Park, J., Pereira, T.M.D., Reardon, K., Savage, S., Sipőcz, B.M., Stansby, D., Jain, Y., Taylor, G., Yadav, T., Rajul, D.T.K., Sunpy Contributors: 2020, The SunPy project: open source development and status of the version 1.0 core package. *Astrophys. J.* **890**(1), 68. DOI. ADS.

[CR41] Virtanen, P., Gommers, R., Oliphant, T.E., Haberland, M., Reddy, T., Cournapeau, D., Burovski, E., Peterson, P., Weckesser, W., Bright, J., Van Der Walt, S.J., Brett, M., Wilson, J., Millman, K.J., Mayorov, N., Nelson, A.R.J., Jones, E., Kern, R., Larson, E., Carey, C.J., Polat, İ., Feng, Y., Moore, E.W., VanderPlas, J., Laxalde, D., Perktold, J., Cimrman, R., Henriksen, I., Quintero, E.A., Harris, C.R., Archibald, A.M., Ribeiro, A.H., Pedregosa, F., Van Mulbregt, P., SciPy 1.0 Contributors, Vijaykumar, A., Bardelli, A.P., Rothberg, A., Hilboll, A., Kloeckner, A., Scopatz, A., Lee, A., Rokem, A., Woods, C.N., Fulton, C., Masson, C., Häggström, C., Fitzgerald, C., Nicholson, D.A., Hagen, D.R., Pasechnik, D.V., Olivetti, E., Martin, E., Wieser, E., Silva, F., Lenders, F., Wilhelm, F., Young, G., Price, G.A., Ingold, G.-L., Allen, G.E., Lee, G.R., Audren, H., Probst, I., Dietrich, J.P., Silterra, J., Webber, J.T., Slavič, J., Nothman, J., Buchner, J., Kulick, J., Schönberger, J.L., De Miranda Cardoso, J.V., Reimer, J., Harrington, J., Rodríguez, J.L.C., Nunez-Iglesias, J., Kuczynski, J., Tritz, K., Thoma, M., Newville, M., Kümmerer, M., Bolingbroke, M., Tartre, M., Pak, M., Smith, N.J., Nowaczyk, N., Shebanov, N., Pavlyk, O., Brodtkorb, P.A., Lee, P., McGibbon, R.T., Feldbauer, R., Lewis, S., Tygier, S., Sievert, S., Vigna, S., Peterson, S., More, S., Pudlik, T., Oshima, T., Pingel, T.J., Robitaille, T.P., Spura, T., Jones, T.R., Cera, T., Leslie, T., Zito, T., Krauss, T., Upadhyay, U., Halchenko, Y.O., Vázquez-Baeza, Y.: 2020, SciPy 1.0: fundamental algorithms for scientific computing in Python. *Nat. Methods* **17**(3), 261. DOI. ADS. 32015543 10.1038/s41592-019-0686-2 PMC7056644

[CR42] Waskom, M.: 2021, seaborn: statistical data visualization. *J. Open Sour. Softw.* **6**(60), 3021. DOI. ADS.

[CR43] Zhang, J., Wang, Y., Liu, Y.: 2010, Statistical properties of solar active regions obtained from an automatic detection system and the computational biases. *Astrophys. J.* **723**(2), 1006. DOI. ADS.

